# Phase engineering of covalent triazine frameworks to enhance photocatalytic hydrogen evolution performance†

**DOI:** 10.1039/d4sc06496h

**Published:** 2025-01-28

**Authors:** Peng Wu, Jijun Lu, Fengshuo Xi, Xiufeng Li, Wenhui Ma, Fangyuan Kang, Shaoyuan Li, Zhongqiu Tong, Qichun Zhang

**Affiliations:** a Faculty of Metallurgical and Energy Engineering, Kunming University of Science and Technology Kunming 650093 China; b School of Engineering, Yunnan University Kunming 650500 China; c Department of Materials Science and Engineering, City University of Hong Kong Tat Chee Avenue 83, Kowloon Hong Kong SAR 999077 P. R. China; d Department of Chemistry, Center of Super-Diamond and Advanced Films (COSDAF) & Hong Kong Institute of Clean Energy (HKICE), City University of Hong Kong Kowloon Hong Kong SAR 999077 P. R. China

## Abstract

Photocatalytic water splitting for hydrogen production has been considered as an effective approach to address the current energy crisis and environmental challenges. Among all materials for such applications, covalent triazine frameworks (CTFs) are regarded as ideal candidates owing to their conjugated structures with rich aromatic nitrogen atoms, which can provide abundant active sites, suitable bandgaps, good structural tunability, and high chemical stability. Although current research studies have shown that the modification of functional groups in CTFs can adjust the band structure and carrier flow characteristics of photocatalysts, leading to improved performance, the impact of the intrinsic structural characteristics of CTFs (*e.g.*, stacking modes, hydrogen bonding) on their photocatalytic performance remains unclear. In this paper, we demonstrate that the photocatalytic hydrogen evolution performance of CTFs can be enhanced through tuning their stacking arrangement, because the stacking modes affect the bandgaps of materials as well as their carrier separation and transfer efficiency. Under visible light conditions, CTF-AA (AA stacking) exhibited a hydrogen evolution rate of 4691.73 μmol g^−1^ h^−1^, which is 37.4% higher than that of CTF-AB (AB stacking, 3415.30 μmol g^−1^ h^−1^). Clearly, the stacking modes significantly influence the cycling stability of CTFs. After eight cycles (over 32 h), CTF-AA maintains its photocatalytic activity and initial performance with a slight decline, while CTF-AB only retains 56.8% of its initial hydrogen evolution rate. Theoretical calculations and physical characterization confirm that the transition of the stacking mode from AB to AA enhances interlayer overlapping, increases the energy level of the lowest unoccupied molecular orbital, and improves the separation and mobility of carriers. These combined factors significantly enhance the photocatalytic performance of CTF-AA. This work offers new insights into the relationship between the photocatalytic performance of CTFs and their stacking patterns, providing new guidelines for designing CTF catalysts with improved activity.

## Abstract 2

This paper examines how stacking modes affect the photocatalytic performance of covalent triazine frameworks (CTFs), revealing that AA stacking enhances hydrogen evolution by 37.4% compared to AB stacking and improves cycling stability.

## Graphical abstract

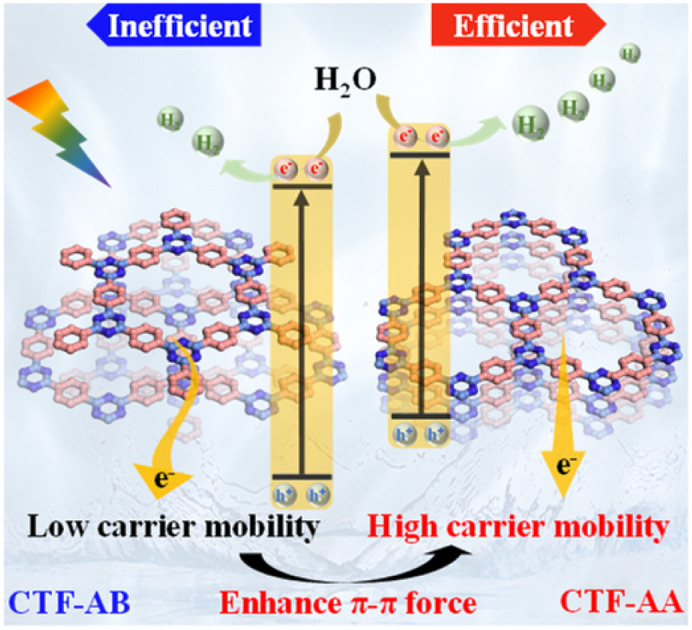

## Introduction

Photocatalytic water splitting to produce hydrogen has been widely considered as an effective approach to address energy crisis, reduce CO_2_ emission and solve green-house issues.^1–3^ Recent advancements indicate that several semiconducting materials, including metal oxides,^4^ metal sulfides,^5^ and metal nitrides,^6^ show excellent performance. However, these traditional inorganic semiconductor materials face several challenges, such as severe recombination of photogenerated electron–hole pairs, inadequate charge separation pathways, and material photocorrosion, which significantly restrict their large-scale applications.^7–9^ These negative factors strongly inspire scientists to switch to porous organic polymer semiconductors due to their large surface area, broad light absorption range, and tunable structure and functionality.^10^ In photocatalytic water splitting, materials such as graphitic carbon nitride (g-C_3_N_4_),^11^ covalent triazine frameworks (CTFs),^12^ and conjugated microporous polymers^13^ exhibit excellent performance.

Compared to other materials, CTFs have received significant attention in photocatalytic water splitting for hydrogen production owing to their fully conjugated structures with rich aromatic nitrogen atoms as active sites, tunable band structures, strong visible light response, and high chemical stability.^14,15^ However, several critical issues still need to be addressed in this field, including limited light absorption range, low charge transfer efficiency, and the catalytic activity.^16^ Designing new molecular structures has emerged as an effective strategy for tuning band structures, enhancing carrier mobility and separation capabilities, and improving photocatalytic hydrogen evolution performance.^17,18^ For example, Wang *et al.* developed ultrathin crystalline amide-functionalized CFT nanosheets through a redox exfoliation strategy. Experimental and theoretical analyses revealed that introducing amide groups as electron donors into CTFs could optimize their band structure and enhance their visible light response, thereby improving photocatalytic performance.^19^ Li *et al.* employed a simple ball milling and acidification method to introduce chlorine into the interlayers of CTF-1, resulting in Cl-intercalated CTF-1 (Cl-ECF) with a distinctive electronic structure. This modification facilitated charge transfer and enhanced the photocatalytic hydrogen evolution performance of Cl-ECF.^20^ Sun *et al.* demonstrated that aromatic molecules could be efficiently and stably adsorbed onto the porous conjugated molecular planes of two-dimensional triazine-based polymers (2D-tp) through π–π stacking interactions. This surface molecular doping effect optimized the band structure, enhanced the separation of photo-generated charge carriers, and resulted in a ∼23.6-fold increase in the photocatalytic hydrogen evolution rate.^21^ The study indicated that the mobility of carriers between adjacent layers of CTFs significantly influenced their photocatalytic hydrogen evolution performance.

The stacking structure is a vital characteristic of CTFs. Theoretical studies have indicated that the stacking pattern significantly influences the optical properties, electronic structure, and band structure of CTFs,^22^ which further affect their photocatalytic hydrogen evolution performance.^23–25^ However, the impact of stacking modes on photocatalytic hydrogen evolution performance and its underlying mechanisms remains unclear. In this study, we synthesized CTFs with different stacking patterns and investigated their effects on photocatalytic H_2_ evolution performance through both experiments and theoretical calculations. Photocatalytic water splitting experiments were conducted under visible light conditions, and the results revealed that the AA stacking CTF-1 (CTF-AA) exhibited higher photocatalytic activity with a hydrogen evolution rate of 4691.73 μmol g^−1^ h^−1^, which was ∼37.4% greater than that of the AB stacking CTF-1 (CTF-AB, 3415.30 μmol g^−1^ h^−1^). Additionally, after eight testing cycles (over 32 hours), CTF-AA still maintained its photocatalytic activity, while the photocatalytic performance of CTF-AB decreased to 56.8% of its original hydrogen evolution rate. Physical characterization and theoretical calculations confirmed that CTF-AA exhibited stronger interlayer conjugation than CTF-AB. This resulted in a higher lowest unoccupied molecular orbital (LUMO) position and enhanced the separation and mobility of charge carriers, contributing to the superior performance of CTF-AA in photocatalytic water splitting for hydrogen production.

## Results and discussion

**Fig. 1:**
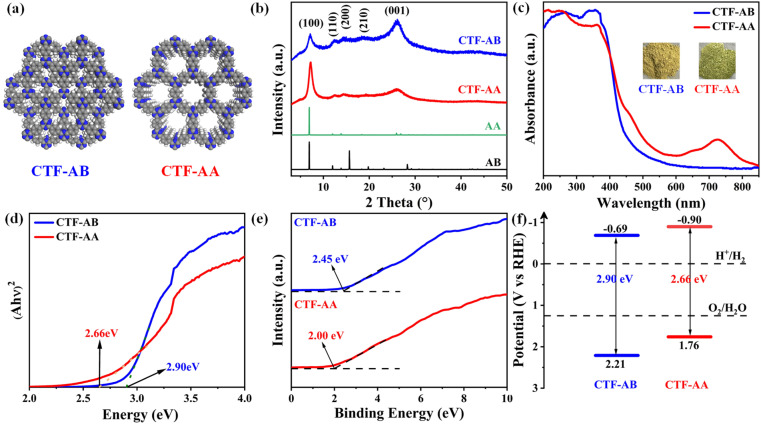
(a) The chemical structure of CTF-1 in AB stacking and AA stacking models (C, gray; N, blue; and H, white). (b) Experimental and simulated PXRD pattern of CTF-AB and CTF-AA based on DFT-D3 optimized structures. (c) UV-visible diffuse reflectance spectrum, (d) plot of transformed Kubelka–Munk function against photon energy, (e) valence band X-ray photoelectron spectroscopy and (f) band structure diagram of CTF-AB and CTF-AA.

CTF-AB with a triazine structural unit as the node and an AB stacking mode was synthesized through a one-step transformation method using trifluoromethanesulfonic acid (CF_3_SO_3_H) as a catalyst.^26^ However, CTF-AA with an AA stacking structure was directly obtained through a low-temperature microwave-assisted synthesis strategy under nearly identical reaction conditions.^27^ The structural model of CTF-1 with both AA and AB stacking patterns is shown in Fig. 1a. Crystal structure characteristics of CTF-AB and CTF-AA were investigated through powder X-ray diffraction (PXRD). The PXRD patterns (Fig. 1b) of both CTF-AB and CTF-AA exhibited strong diffraction peaks, indicating well-ordered crystalline structures with hexagonal stacking pores. Notably, a strong diffraction peak at 2*θ* = 7.12° corresponded to the in-plane reflection of the (100) crystal plane. Additionally, three weaker diffraction peaks at 12.38°, 14.29°, and 19.06° can be attributed to the reflections of the (110), (200), and (210) crystal planes, respectively. The diffraction peak at 26.05° corresponded to the (001) crystal plane, representing the interlayer spacing between stacked layers and confirming the π–π stacking in both CTF-AB and CTF-AA.^28^ The diffraction patterns of CTF-AB and CTF-AA were consistent with the patterns predicted by the simulated staggered AB stacking model and ordered AA stacking model, respectively. Moreover, the successful polymerization of CTF-AB and CTF-AA was confirmed *via* Fourier-transform infrared (FT-IR) spectroscopy. A comparison of the infrared spectra of the reactant monomer (1,4-diaminobenzene, DCB) with those of CTF-AB and CTF-AA revealed significant differences (Fig. S1†). The characteristic peak at 2229 cm^−1^ associated with the nitrile group nearly disappeared, indicating that the nitrile group was effectively involved in the reaction under CF_3_SO_3_H catalysis. Moreover, three distinct strong absorption peaks at 802, 1348, and 1509 cm^−1^ corresponded to the breathing vibration of the triazine unit, C=N stretching vibration, and C–N stretching vibration, respectively.^14,29,30^ These characteristic peaks indicate the formation of the triazine ring and confirm the completion of the synthesis reactions for CTF-AB and CTF-AA under mild reaction conditions. However, a detailed comparison of the infrared spectra of CTF-AB and CTF-AA revealed significant differences. The infrared spectrum of CTF-AB exhibited numerous additional peaks, which may result from unreacted or partially reacted compounds (including DCB, CF_3_SO_3_H, and intermediate polymers). These compounds might be trapped in the disordered stacking interlayers of CTF-AB, making them difficult to remove through simple treatments. In contrast, the infrared spectrum of CTF-AA exhibited fewer additional peaks, indicating a higher purity of CTF-AA.

The elemental compositions of CTF-AB and CTF-AA were analyzed *via* X-ray photoelectron spectroscopy (XPS). The results (Fig. S2a†) revealed that CTF-AB contained C, N, O, and F elements, suggesting the residual presence of CF_3_SO_3_H in its framework. In contrast, CTF-AA exhibited minimal levels of F and O, indicating a near-complete removal of residual CF_3_SO_3_H. Further analysis of the high-resolution XPS spectra of both materials revealed distinct features. The high-resolution C 1s XPS spectrum of CTF-AB (Fig. S2b†) exhibited two prominent peaks at 284.8 and 286.9 eV, corresponding to carbon in the benzene ring and triazine ring, respectively. Additionally, a peak at 288.8 eV can be attributed to C–F bonds. The high-resolution N 1s XPS spectrum of CTF-AB (Fig. S2c†) exhibited a nitrogen signal at 398.8 eV, which corresponded to the triazine ring. In contrast, the C 1s spectrum of CTF-AA (Fig. S2d†) exhibited peaks at 284.8 and 287.0 eV, which were associated with the benzene ring and triazine ring, respectively. Similarly, the N 1s spectrum (Fig. S2e†) displayed a peak at 398.8 eV with few impurity peaks, corresponding to the nitrogen in the triazine ring. This observation further confirmed the high purity of CTF-AA.

A series of spectroscopic characterization studies were conducted to further analyze the photoelectric properties and band gap structures of CTF-AB and CTF-AA. The UV-visible (UV-vis) diffuse reflectance spectra (Fig. 1c) revealed that both CTF-AB and CTF-AA exhibited strong absorption in the visible light range, with absorption edges at 468 and 578 nm, respectively. Unlike CTF-AB, CTF-AA exhibited a strong absorption peak in the near-infrared region (∼725 nm) owing to the higher interlayer conjugation resulting from the AA stacking mode.^31–33^ The Tauc-plot spectra (Fig. 1d) revealed bandgaps of 2.90 and 2.66 eV for CTF-AB and CTF-AA, respectively. The valence band maximum (VBM) energy levels for CTF-AB and CTF-AA were determined through valence band X-ray photoelectron spectroscopy (VB-XPS) (Fig. 1e). CTF-AB and CTF-AA exhibited VBM levels of 2.45 and 2.00 eV, respectively. The standard hydrogen electrode potentials (*E*_VB, NHE_) of both materials were calculated using the formula: *E*_(VB, NHE)_ = *φ* + *E*_(VB, XPS)_ − 4.44, where *φ* represents the work function of the instrument (4.2 eV).^34,35^ As shown in Table S1,† the CTF-AB and CTF-AA featured valence band maxima (*E*_VB_) of 2.21 and 1.76 eV, respectively. The conduction band minima (*E*_CB_) of CTF-AB and CTF-AA were calculated to be −0.69 and −0.90 eV, respectively, using band structure calculation formulae. These *E*_CB_ values were consistent with the flat-band potentials estimated from the Mott–Schottky tests (Fig. S3†). Fig. 1f shows the specific bandgap structures of CTF-AB and CTF-AA. The comparison of these bandgap structures revealed that CTF-AA had a narrower bandgap and a more negative conduction band than CTF-AB. The narrower bandgap suggested that CTF-AA had a shorter carrier migration pathway and higher charge separation efficiency. The more negative conduction band position indicated a stronger reduction potential, which facilitated subsequent photocatalytic hydrogen reduction reactions and enhanced photocatalytic activity.

**Fig. 2:**
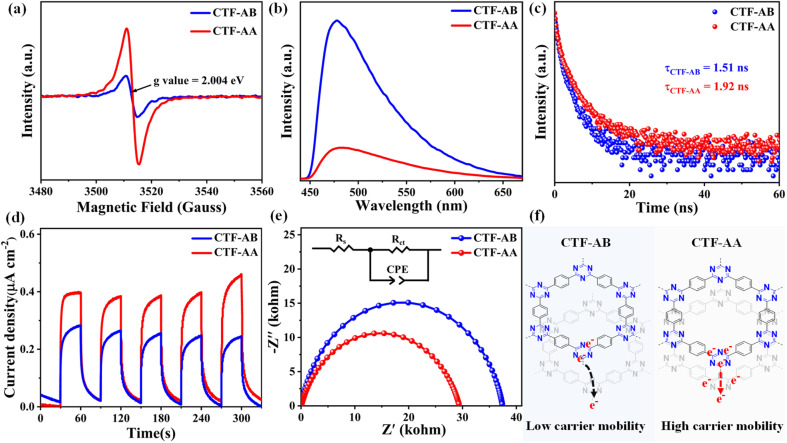
(a) EPR spectra, (b) steady-state photoluminescence spectra (excitation wavelength: 410 nm), (c) transient photoluminescence spectrum (excitation wavelength: 410, PL emission collected from 430 nm to 820 nm during TRPL measurements), (d) transient photocurrent response curves, (e) EIS Nyquist plots of CTF-AB and CTF-AA, and (f) schematic representation of the charge carrier mobility comparison between CTF-AB and CTF-AA.

The concentration of unpaired electrons in both CTF-AB and CTF-AA was determined *via* electron paramagnetic resonance (EPR) spectroscopy. The EPR spectra, recorded in the absence of light, revealed a Lorentzian peak centered at a *g* value of 2.004 (Fig. 2a), which can be attributed to unpaired electrons on the π-conjugated aromatic rings of the materials. Notably, CTF-AA exhibited a stronger EPR signal than CTF-AB, indicating a higher concentration of unpaired electrons. This enhancement in the signal can be explained by the effect of the stacking mode on the electronic structure. In CTF-AA, the tighter interlayer arrangement and enhanced π–π interactions lead to easier localization of electrons in certain regions. The crystallographic characterization indicates that CTF-AA has a smaller interlayer distance, and the nitrogen–carbon octahedra are more effectively overlapped in space, which facilitates the overlap of electron clouds between adjacent layers and promotes the delocalization of electrons across the stacked layers. Therefore, the stronger EPR signal in CTF-AA is likely associated with higher electron density and more efficient charge carrier generation and transport, which contribute to better charge separation during the photocatalytic process.^36^ To investigate the photogenerated charge separation characteristics, photoluminescence (PL) spectra and time-resolved PL (TRPL) decay measurements were conducted. The PL spectra (Fig. 2b) of both samples revealed strong fluorescence emission bands at 478 nm. CTF-AB exhibited a pronounced fluorescence signal, while the fluorescence signal intensity of CTF-AA was only one-fifth that of CTF-AB, indicating a lower photogenerated charge recombination rate of CTF-AA. The reduced fluorescence intensity of CTF-AA is directly linked to its ability that can efficiently separate photogenerated electron–hole pairs, thereby reducing the likelihood of recombination and promoting better photocatalytic performance. To estimate exciton recombination, time-resolved fluorescence decay spectra were recorded for both materials, revealing average lifetimes of 1.92 ns for CTF-AB and 1.51 ns for CTF-AA (Fig. 2c and Table S2†). CTF-AA exhibited a longer lifetime of photogenerated carriers than CTF-AB, indicating more efficient charge separation and reduced recombination, which contributes to its higher photocatalytic hydrogen production efficiency. The observed difference in carrier lifetime between CTF-AB and CTF-AA can be attributed to their distinct structural and electronic properties. CTF-AA with higher electron density likely exhibits more efficient charge transfer and reduced recombination, contributing to its superior photocatalytic performance. In contrast, CTF-AB with lower electron density may suffer from greater recombination losses, leading to shorter carrier lifetimes and less efficient photocatalytic performance.

To evaluate the photoelectrical response capabilities of these CTFs, photocurrent tests were performed in a 0.2 M sodium sulfate solution (pH = 6.8), with CTFs deposited on indium tin oxide substrates. The instantaneous photocurrent responses during several on–off light irradiation cycles revealed that CTF-AA exhibited a stronger photocurrent response signal than CTF-AB (Fig. 2d). This result indicates that the AA stacking structure enabled more effective spatial separation of photogenerated carriers than the AB stacking structure. To further investigate the charge separation and transport properties of both materials, electrochemical impedance spectroscopy (EIS) was conducted. The charge transfer resistance (*R*_ct_) decreased from 37.4 kΩ for CTF-AB to 29.4 kΩ for CTF-AA (Fig. 2e and Table S3†), suggesting that CTF-AA had a higher rate of photogenerated carrier transfer than CTF-AB. The experimental results (Fig. 2f) revealed that the transition from AB stacking patterns to AA stacking modes endowed the CTF layers with a more stable π-conjugated structure, leading to a higher concentration of unpaired electrons in CTF-AA. These structural changes improved the ability of CTF-AA to effectively separate and transfer carriers.

**Fig. 3:**
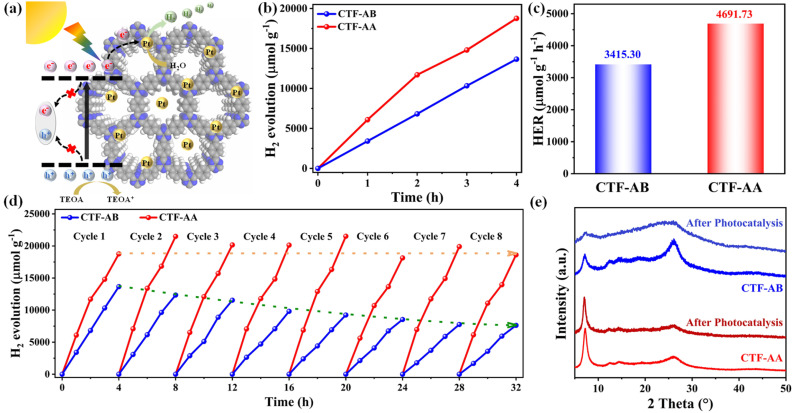
(a) The process of photocatalytic water splitting for hydrogen production in CTFs. (b) Photocatalytic hydrogen evolution performance plots, (c) comparison of hydrogen evolution rates and (d) long-term cycling stability test for photocatalytic hydrogen production of CTF-AB and CTF-AA; (e) PXRD analysis of CTF-AB and CTF-AA before and after a long-term cycling photocatalytic test.

Moreover, the photocatalytic hydrogen production performance of CTF-AB and CTF-AA was evaluated under simulated visible light (*λ* > 420 nm) in an aqueous solution containing triethanolamine (TEOA) as a sacrificial agent and a small amount of Pt as the co-catalyst (3 wt%) (Fig. 3a). Pt nanoparticles were incorporated into CTFs through *in situ* photodeposition.^37^Fig. 3b and c display the photocatalytic hydrogen evolution performance of CTF-AB and CTF-AA. These results revealed a strong linear correlation between the molar amount of hydrogen produced and time for both CTF-AB and CTF-AA. CTF-AA exhibited a hydrogen evolution rate of 4691.73 μmol g^−1^ h^−1^, which was ∼37.4% higher than the rate of CTF-AB (3415.30 μmol g^−1^ h^−1^). During long-term cycling stability tests (Fig. 3d), CTF-AA exhibited excellent cycling stability and maintained stable hydrogen evolution performance across eight test cycles with minimal decay. In contrast, CTF-AB exhibited poor cycling stability, retaining only 56.8% of its initial capacity after eight cycles. The PXRD analysis of CTF-AB and CTF-AA after cycling stability testing (Fig. 3e) revealed that CTF-AB exhibited significant structural changes, indicated by a notable decrease in diffraction peak intensity, suggesting the disruption of its crystalline structure. In contrast, the PXRD pattern of CTF-AA displayed no significant changes, indicating its superior structural stability. These results indicate that CTF-AA exhibited excellent photocatalytic hydrogen production performance and higher structural stability than CTF-AB. This suggests that the stacking modes of CTFs significantly affect their photocatalytic efficiency and structural stability.

**Fig. 4:**
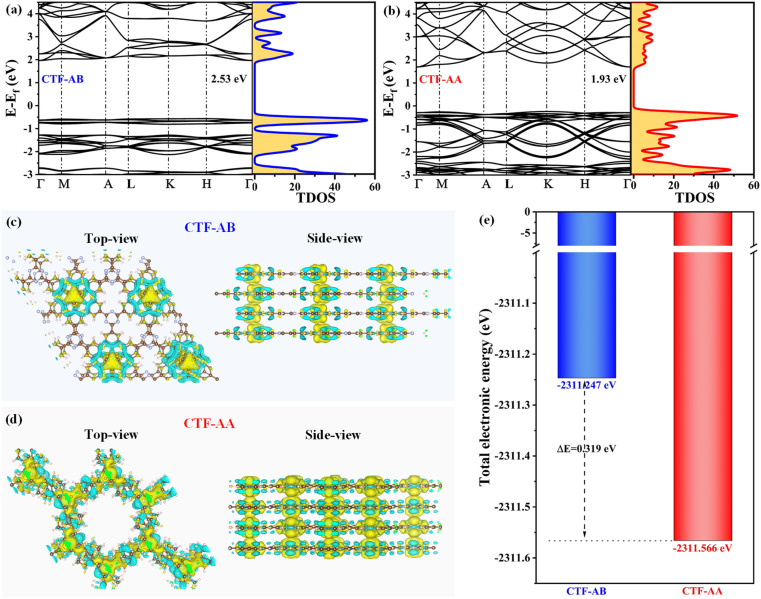
Band structures and total density of states (TDOS) are shown for CTF-AB (a) and CTF-AA (b). Differential charge density distribution is depicted in top view and side views for CTF-AB (c) and CTF-AA (d) (yellow indicating increased charge density and cyan indicating decreased charge density). (e) Total electronic energy comparison between CTF-AB and CTF-AA.

The effect of stacking structures on the photocatalytic hydrogen evolution performance of CTFs was investigated through density functional theory (DFT) calculations. This study examined the band structure, electronic structure, and charge density. The band structure and total density of states for CTF-AB and CTF-AA were calculated through the generalized gradient approximation method (Fig. 4a and b). Both CTF-AB and CTF-AA were identified as direct bandgap semiconductors based on the high-symmetry point path in the Brillouin zone.^38^ CTF-AB and CTF-AA exhibited bandgaps of 2.53 and 1.93 eV, respectively. Additionally, CTF-AB exhibited more concentrated energy levels, while CTF-AA displayed more continuous energy levels. The continuous energy levels in CTF-AA suggested a more uniform electron distribution, promoting easier electron movement. On the other hand, the concentrated energy levels can lead to the increased electron–hole recombination, potentially reducing photocatalytic activity.^39,40^ The computed highest occupied molecular orbital (HOMO) and LUMO of CTF-AB and CTF-AA (Fig. S4†) indicated that the HOMO orbital mainly comprised π-bonds from benzene rings and lone pairs of nitrogen atoms, while the LUMO orbital was mainly derived from the triazine rings. The triazine rings served as the primary reactive sites, participating in photocatalytic reduction reactions to produce hydrogen.

Differential charge density calculations provided insights into the impact of stacking modes on the charge distribution and electron transfer behavior in CTFs (Fig. 4c and d). The comparison of the top and side views of charge density differences between CTF-AB and CTF-AA revealed that CTF-AA had more sites with significant charge density variations and tighter interlayer electronic cloud overlap than CTF-AB, which is favourable for electron transfer. This indicates that CTF-AA exhibited higher charge carrier transfer efficiency and stronger interlayer interactions.^21,29,41^ The total energy and energy difference (Δ*E*) of CTF-AB and CTF-AA at the same layer number were calculated using the DFT-D3 level method (Fig. 4e). CTF-AB and CTF-AA exhibited total electronic energies of −2311.247 and −2311.566 eV, respectively, yielding an energy difference of 0.319 eV (∼30.8 kJ mol^−1^). This indicates that CTF-AA was more stable than CTF-AB. Therefore, CTF-AA exhibited stronger interlayer interactions than CTF-AB, resulting in superior structural stability during the photocatalytic process and lower susceptibility of CTF-AA to degradation.

**Fig. 5:**
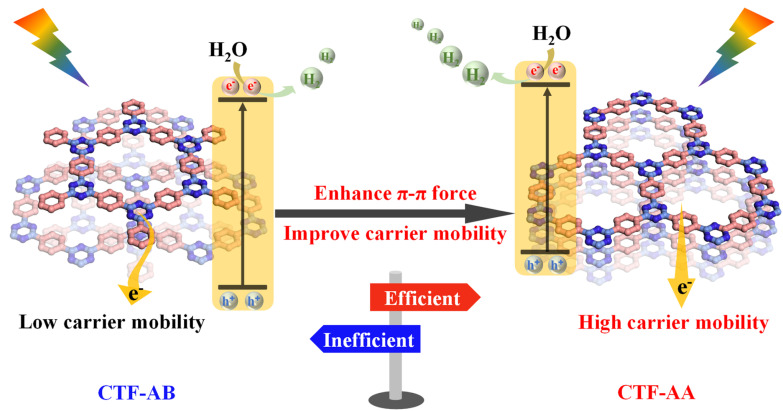
Enhancement mechanism of photocatalytic hydrogen evolution performance of CTF-AA.

Fig. 5 illustrates the effect of changes in stacking structures on the interlayer conjugation, band structure, and electronic and optical properties of CTF-1. Both experimental and theoretical results indicated that the overlapping stacking pattern in CTF-AA exhibited a higher degree of interlayer conjugation than the interleaved stacking mode in CTF-AB. AA stacking enhanced the light absorption capacity of CTF-AA and led to a narrower bandgap. Consequently, CTF-AA exhibited stronger hydrogen evolution reduction potential during the photocatalytic process, provided more favorable pathways for photoinduced charge carrier transport, and featured higher rates of charge carrier separation and mobility, thereby improving its photocatalytic hydrogen evolution performance. Additionally, the stronger interlayer interactions endowed CTF-AA with excellent cycling stability in hydrogen evolution, further enhancing its potential for practical applications.

## Conclusions

In summary, this study comprehensively investigates the potential of CTFs in photocatalytic water-splitting under visible light, particularly focusing on the comparison between AB stacking and AA stacking. The results revealed that CTF-AA exhibited higher photocatalytic activity, with a hydrogen evolution rate of 4691.73 μmol g^−1^ h^−1^, which was 37.4% higher than that of CTF-AB (3415.30 μmol g^−1^ h^−1^). Additionally, after eight testing cycles (over 32 h), CTF-AA still maintained its photocatalytic activity with a slight decline, while CTF-AB only retained 56.8% of its initial hydrogen evolution performance. Both experimental and theoretical calculations revealed that CTF-AA had a narrower bandgap and a higher hydrogen evolution reduction potential. During photocatalysis, the higher interlayer overlapping and narrower bandgap of CTF-AA enhanced the transport, separation, and mobility of charge carriers, making CTF-AA more effective for hydrogen evolution compared with CTF-AB. Moreover, the stronger interlayer interactions in CTF-AA contributed to its superior structural stability and cycling stability of hydrogen evolution. The findings of this study underscore the critical impact of stacking structures on photocatalytic performance, indicating that optimizing these structures and synthesis methods can significantly enhance the efficacy of photocatalysts. This discovery provides new directions for the design of more active CTF photocatalysts, highlighting the necessity of further research on material structure and functionality to address future challenges in renewable energy.

## Data availability

The data that support the findings of this study are available in the ESI† of this article.

## Author contributions

Peng Wu: methodology, investigation, data analysis and writing – original draft. Jijun Lu: investigation, data analysis. Fengshuo Xi: investigation. Xiufeng Li: investigation. Wenhui Ma: investigation, resources. Fangyuan Kang: investigation. Shaoyuan Li: conceptualization, writing – review and editing, funding acquisition and supervision. Zhongqiu Tong: conceptualization, writing – review and editing and supervision. Qichun Zhang: conceptualization, writing – review and editing, and supervision.

## Conflicts of interest

There are no conflicts to declare.

## Supplementary Material

SC-OLF-D4SC06496H-s001
