# Supplementary material for: Phase engineering of covalent triazine frameworks to enhance photocatalytic hydrogen evolution performance
Source: Chem Sci. 2025 Jan 28;16(9):4127–35. doi: 10.1039/d4sc06496h (PMC11788819; doi:10.1039/d4sc06496h)
Supplement: SC-016-D4SC06496H-s001 [file SC-016-D4SC06496H-s001.pdf]

Supplementary Information (SI) for Chemical Science.
This journal is © The Royal Society of Chemistry 2025

Supporting Information

Phase Engineering of Covalent Triazine Frameworks to Enhance Photocatalytic Hydrogen Evolution Performance

Peng Wu,[a] Jijun Lu,[a] Fengshuo Xi,[a] Xiufeng Li,[a] Wenhui Ma,[b] Fangyuan Kang,[c] Shaoyuan Li,*[a] Zhongqiu Tong*[a] and Qichun Zhang*[cd]

[a] Faculty of Metallurgical and Energy Engineering, Kunming University of Science and Technology, Kunming, 650093, China.
[b] School of Engineering, Yunnan University, Kunming 650500, China.
[c] Department of Materials Science and Engineering, City University of Hong Kong, Tat Chee Avenue 83, Kowloon, Hong Kong, SAR, 999077 P. R. China.
[d] Department of Chemistry, Center of Super-Diamond and Advanced Films (COSDAF) & Hong Kong Institute of Clean Energy (HKICE), City University of Hong Kong, Kowloon, Hong Kong, SAR, 999077 P. R. China.

Experimental Section

Experimental Reagents: In this experiment, trifluoromethanesulfonic acid (CF_3SO_3H) and 1,4-dicyanobenzene (DCB) were obtained from Shanghai Macklin Biochemical Co., Ltd. Tetrahydrofuran (THF) and chloroplatinic acid hydrate ($H_2PtCl_6 \cdot 6H_2O$) were purchased from Aladdin Reagent Co., Ltd. (Shanghai, China). Triethanolamine (TEOA) was obtained from Sigma-Aldrich, and acetone was purchased from Chongqing Chuandong Chemical (Group) Co., Ltd. Deionized water was used throughout all experiments. All chemicals were used as received without further treatment unless specified otherwise.

Preparation of CTF-AB Materials: First, 0.256 g (2 mmol) of DCB monomer and 0.15 g (1 mmol) of CF_3SO_3H were added to a Pyrex tube. After the thorough mixing of these materials, the tube was sealed under vacuum and cooled with liquid nitrogen. The tube was then placed in a muffle furnace and heated to 250 °C at a rate of 5 °C min^{-1} for 12 h. After the reaction, the Pyrex tube was cooled with liquid nitrogen to reduce the system pressure. The resulting powder was sequentially rinsed with an NH_3 aqueous solution, ethanol, acetone, and THF. The sample was vacuum-dried at 60 °C for 12 h to obtain a yellow powder. Subsequently, 100 mg of the bulk powder was combined with zirconia balls and 10 mL of dimethylformamide (DMF) in a ball-milling jar. The mixture was ball-milled at 50 Hz for 6 h. Finally, the powder was collected via vacuum filtration to obtain the CTF-AB powder.

Preparation of CTF-AA Materials: First, 0.128 g (1 mmol) of DCB monomer was mixed with 0.225 g (1.5 mmol) of CF_3SO_3H in a Pyrex tube. The tube was sealed under vacuum and cooled with liquid nitrogen. Subsequently, the tube was placed in a domestic microwave oven. The reaction proceeded under microwave irradiation for 20 min at a constant power output of 700 W. After the reaction, the Pyrex tube was cooled with liquid nitrogen to reduce the system pressure. The resulting powder was sequentially rinsed with an NH_3 aqueous solution, ethanol, acetone, and THF. The sample was vacuum-dried at 60°C for 12 h to obtain a yellowish-green powder. Subsequently, 100 mg of bulk powder was combined with zirconia balls and 10 mL of DMF in a ball-milling jar. The mixture was ball-milled at 50 Hz for 6 h. Finally, the powder was collected via vacuum filtration to obtain the CTF-AA powder.

Sample Characterizations: The crystal structures of photocatalysts were analyzed using an X-ray diffractometer (Rigaku SmartLab Corporation, Japan) with a scanning range of 3°–50°. FTIR spectroscopy was performed using the KBr pellet method with

an infrared spectrometer (Bruker ALPHA). The elemental oxidation states on the material surface were examined using an X-ray photoelectron spectrometer (Thermo Fisher Scientific ESCALAB 250Xi). Additionally, the VB structure was analyzed via VB-XPS. The UV–vis diffused reflectance spectra were obtained using a UV–vis spectrophotometer (UV-2600, Japan Shimadzu Co., Ltd). The unpaired electron configuration of the sample was analyzed using an EPR spectrometer (Bruker A300, Bruker Corporation). PL and TRPL emission spectra were recorded using a fluorescence spectrometer (Edinburgh FLS-1000, Edinburgh Instruments).

Photoelectrochemical Measurements: EIS, Mott – Schottky analysis, and transient photocurrent response measurements were conducted using an electrochemical workstation (Shanghai Chenhua CHI650E) equipped with a three-electrode system. A 300 W Xe lamp with an AM-1.5 filter served as the light source. The working electrode was prepared as follows. First, 5 mg of catalyst powder was fully dispersed in a solution of 1 mL anhydrous ethanol and 15 μL of 0.25% Nafion to prepare a suspension. A 5% naphthol solution was used as a binder for the catalysts. Subsequently, 200 μL of the prepared suspension was applied to an ITO glass substrate and subjected to heat treatment on an annealing table to form the working electrode. Photocatalytic measurements were conducted using a 300 W Xe lamp ($\lambda > 420$ nm) as the light source, with a 0.2 M Na_2SO_4 solution as the electrolyte. An Ag/AgCl electrode and a platinum wire were used as the reference electrode and counter electrode, respectively. The Mott–Schottky curves were analyzed using a 0.2 M Na_2SO_4 aqueous solution as the electrolyte, with alternating current frequency rates of 500, 700, and 1000 Hz.

Photocatalytic Performance for Hydrogen Evolution: The photocatalytic hydrogen evolution performance test was conducted using an automatic sampling test system. A 300 W Xe lamp equipped with a 420 nm filter was employed as the light source, with an intensity of 100 mW/cm^2. The reactor had a volume of 100 mL, and the initial pressure was set at 0.08 kPa, while the entire reaction system was maintained in a constant temperature bath at 6 °C. The specific testing steps are as follows: First, 10 mg of the photocatalyst was weighed and ultrasonically dispersed in 45 mL of deionized water. Subsequently, 5 mL of triethanolamine was added as a sacrificial agent, and 3 wt.% of Pt (using $H_2PtCl_6 \cdot 6H_2O$ as the precursor) was loaded via a photoreduction reaction as a co-catalyst. The solution was continuously stirred using a magnetic stirrer to ensure homogeneity. After thorough stirring, the reactor was evacuated to remove dissolved gases completely. The light source was then turned on to initiate the hydrogen

evolution test. To prevent sedimentation of the photocatalyst during the test, continuous stirring was maintained. Gas samples were analyzed hourly using a gas chromatograph over a total duration of 4 h. The hydrogen peak area was measured using the GC7920-TF2A gas chromatograph, and hydrogen production was calculated based on a calibration curve. For the cycling stability test of photocatalytic hydrogen evolution, the same procedures were followed. At the end of each 4-h test cycle, the reaction was halted, and the hydrogen yield was recorded. The photocatalyst was washed with deionized water to remove residual substances and then dried. The above steps were repeated for multiple cycles to evaluate the stability and reproducibility of the photocatalyst. The next test cycle was initiated following the same procedure.

Theoretical Calculation: Density functional theory (DFT) calculations were performed using the Vienna Ab-initio Simulation Package (VASP) program.[1-3] The ionic cores and valence electrons were treated with the projector-augmented wave (PAW) pseudo-potential method alongside a plane-wave basis set.[3, 4] For the electronic exchange-correlation functional, the generalized-gradient approximation (GGA) of Perdew-Burke-Ernzerhof (PBE) was employed.[5] Additionally, the PBE-based DFT-D3 correction was applied to account for Van der Waals interactions.[6] To ensure the consistency and reliability of the results, a uniform plane-wave basis set cutoff of 450 eV and a k-point mesh with a spacing of 0.2 $Å^{-1}$ were utilized. The valence space for the PAW pseudo-potentials included the $2s^22p^2$ and $2s^22p^3$ configurations for carbon and nitrogen, respectively, and the $1s^1$ configuration for hydrogen. All structures were fully relaxed until the Hellmann-Feynman forces on all atoms were below 0.05 eV/Å, and the total energy change was less than 1×10^{-5} eV. The electronic self-consistent iterations for calculating the electronic structure and total energy were considered complete when the energy difference reached 1×10^{-6} eV. The VASPKIT code[7] was employed for the post-processing of VASP output files.

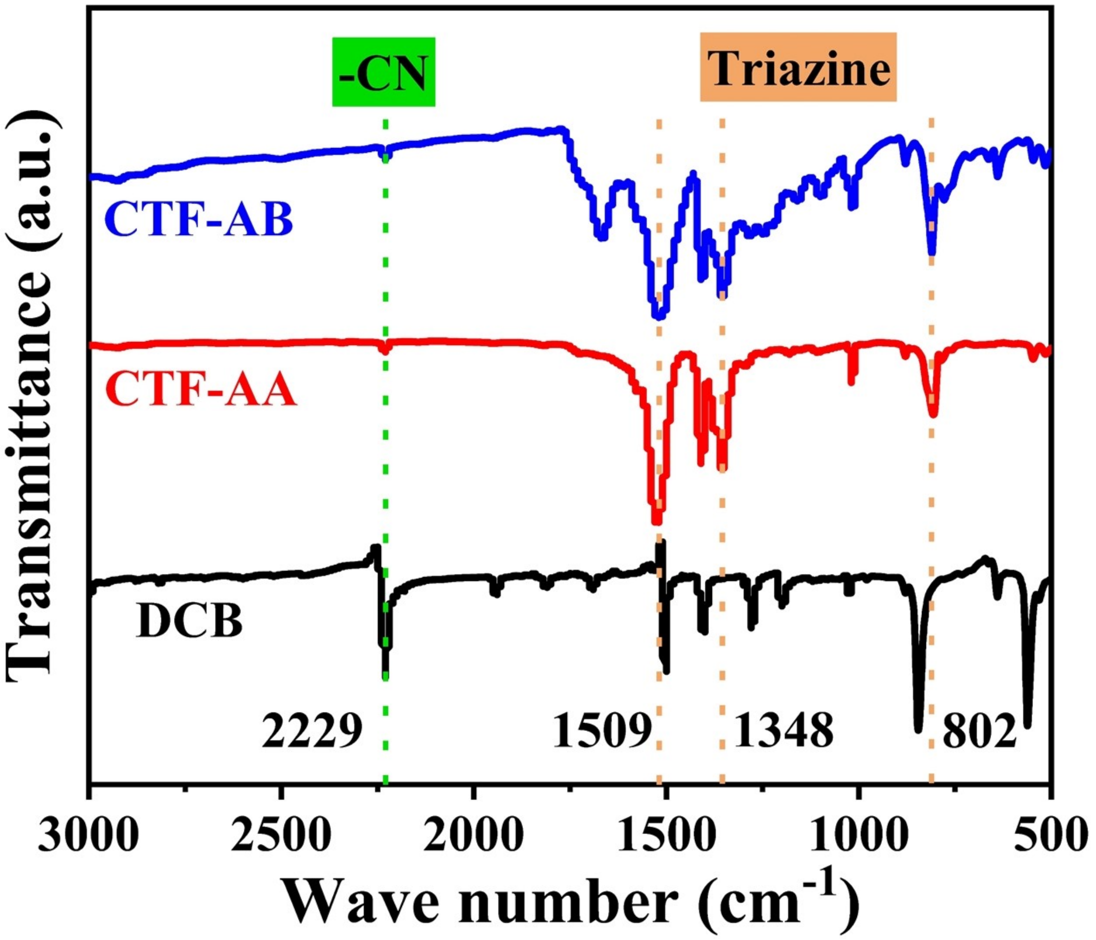

Fig. S1 The FT-IR spectra of CTF-AB, CTF-AA, and 1,4-dicyanobenzene (DCB).

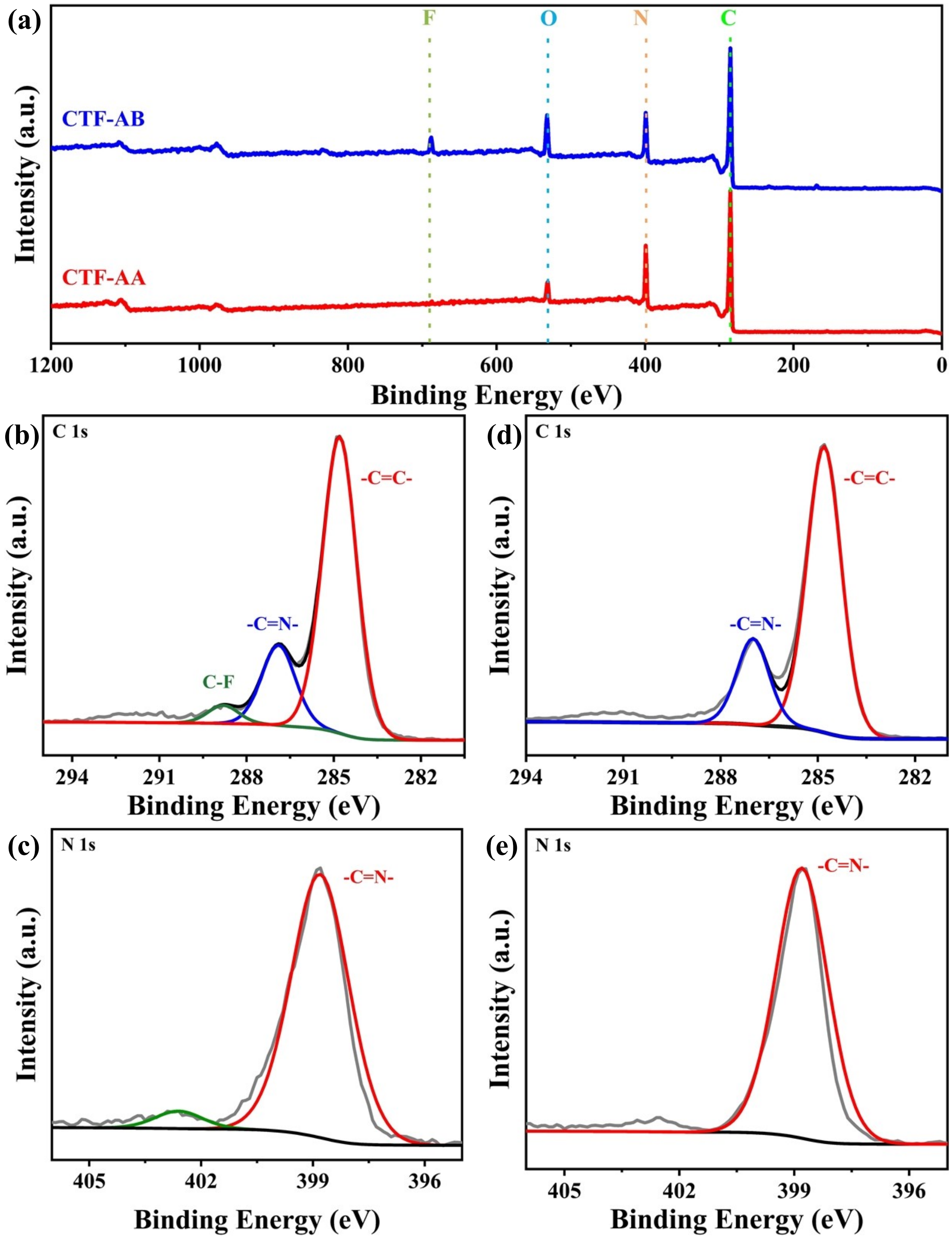

Fig. S2 XPS spectra of CTF-AB (a) survey spectra, (b) high-resolution XPS spectra of C 1s and (c) high-resolution XPS spectra of N 1s; XPS spectra of CTF-AA: (a) survey spectra, (d) high-resolution XPS spectra of C 1s and (e) high-resolution XPS spectra of N 1s.

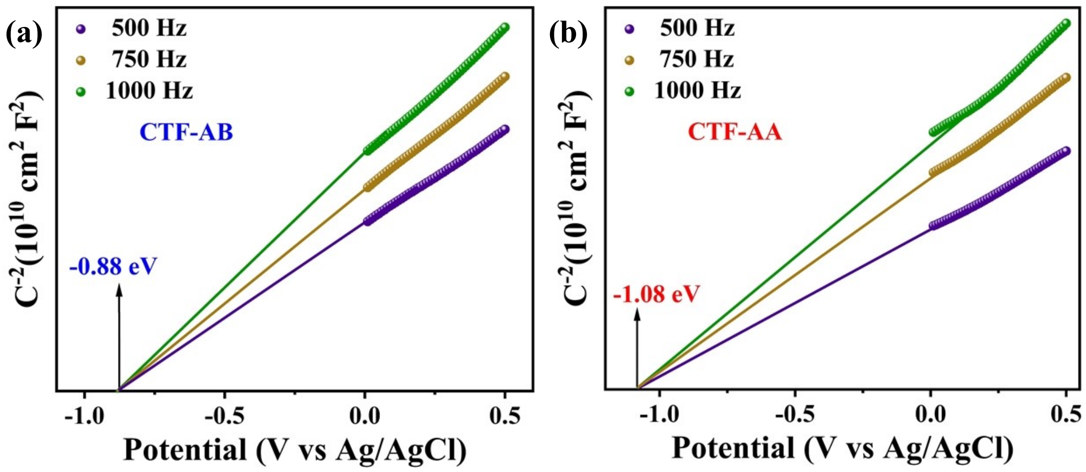

Fig. S3 Mott-Schottky tests for CTF-AB (a) and CTF-AA (b).

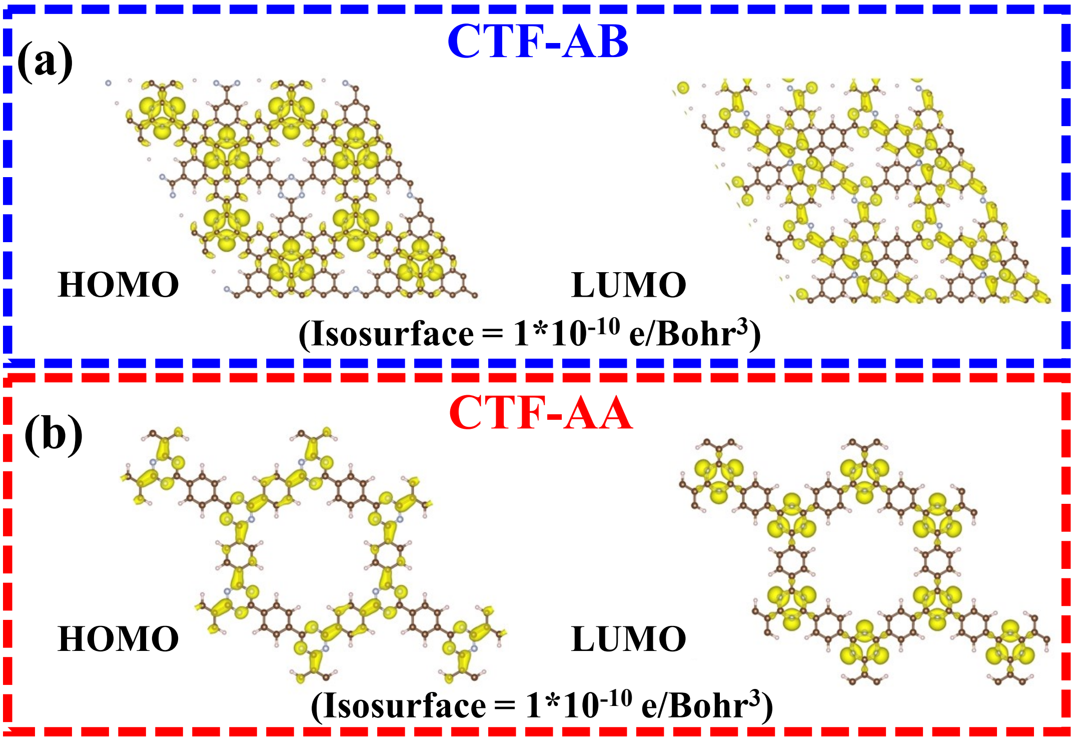

Fig. S4 The partial charge densities of the Highest Occupied Molecular Orbital (HOMO) and Lowest Unoccupied Molecular Orbital (LUMO) for CTF-AB (a) and CTF-AA (b).

Table S1. The calculation process of the standard hydrogen electrode potential ($E_{VB, NHE}$) for CTF-AB and CTF-AA.

Samples	φ	$E_{(VB, XPS)}$	$E_{(VB, NHE)}$
CTF-AB	4.2	2.45	2.21
CTF-AA	4.2	2.00	1.76

Table S2. The TRPL fitting parameters for CTF-AB and CTF-AA.

Samples	τ_1 (ns)	A_1 (%)	τ_2 (ns)	A_2 (%)	τ_{avg} (ns)
CTF-AB	0.6790	4731.3613	2.9470	633.0126	1.51
CTF-AA	0.6657	6374.2852	3.5871	895.9680	1.92

Table S3. The EIS simulated resistance values of CTF-AB and CTF-AA.

Samples	R_s (Ω)	R_{ct} (Ω)
CTF-AB	32.04	37403
CTF-AA	34.56	29383

References:

1 G. Kresse, J. Furthmüller, Efficient iterative schemes for ab initio total-energy calculations using a plane-wave basis set , Phys. Rev. B, 1996, **54**, 11169.

2 G. Kresse, J. Furthmüller. Efficiency of ab-initio total energy calculations for metals and semiconductors using a plane-wave basis set, Compu. Mater. Sci., 1996, **6**, 15-50.

3 G. Kresse, D. Joubert. From ultrasoft pseudopotentials to the projector augmented-wave method, Phys. Rev. B, 1999, **59**, 1758.

4 P. E. Blöchl, Projector augmented-wave method, Phys. Rev. B, 1994, **50**, 17953.

5 J. P. Perdew, K. Burke, M. Ernzerhof, Generalized gradient approximation made simple, Phys. Rev. Lett., 1996, **77**, 3865.

6 S. Grimme, Semiempirical GGA-type density functional constructed with a long-range dispersion correction, J. Compu. Chem., 2006, **27**, 1787-1799.
